# Estimation of co-variance components, genetic parameters, and genetic trends of reproductive traits in community-based breeding program of Bonga sheep in Ethiopia

**DOI:** 10.5713/ajas.20.0413

**Published:** 2020-11-09

**Authors:** Ebadu Areb, Tesfaye Getachew, MA Kirmani, Tegbaru G.silase, Aynalem Haile

**Affiliations:** 1 Bonga Agricultural Research Centre, PO Box 101 Bonga, Ethiopia; 2 International Centre for Agricultural Research in the Dry Area, PO Box 5689, Addis Ababa, Ethiopia; 3 Animal Breeding, Jimma University, PC Depot, Rehmatabad, Srinagar, Jammu and Kashmir, 190017, India

**Keywords:** Bonga Sheep, Genetic Parameter, Genetic Trend, Recording, Reproductive Performance

## Abstract

**Objective:**

The objectives of the study were to evaluate reproductive performance and selection response through genetic trend of community-based breeding programs (CBBPs) of Bonga sheep.

**Methods:**

Reproduction traits data were collected between 2012 and 2018 from Bonga sheep CBBPs. Phenotypic performance was analyzed using the general linear model procedures of Statistical Analysis System. Genetic parameters were estimated by univariate animal model for age at first lambing (AFL) and repeatability models for lambing interval (LI), litter size (LS), and annual reproductive rate (ARR) traits using restricted maximum likelihood method of WOMBAT. For correlations bivariate animal model was used. Best model was chosen based on likelihood ratio test. The genetic trends were estimated by the weighted regression of the average breeding value of the animals on the year of birth/lambing.

**Results:**

The overall least squares mean±standard error of AFL, LI, LS, and ARR were 375± 12.5, 284±9.9, 1.45±0.010, and 2.31±0.050, respectively. Direct heritability estimates for AFL, LI, LS, and ARR were 0.07±0.190, 0.06±0.120, 0.18±0.070, and 0.25±0.203, respectively. The low heritability for both AFL and LI showed that these traits respond little to selection programs but rather highly depend on animal management options. The annual genetic gains were −0.0281 days, −0.016 days, −0.0002 lambs and 0.0003 lambs for AFL, LI, LS, and ARR, respectively.

**Conclusion:**

Implications of the result to future improvement programs were improving management of animals, conservation of prolific flocks and out scaling the CBBP to get better results.

## INTRODUCTION

The total sheep population in Ethiopia was estimated at 31.30 million with 9 breeds [1]. Sheep production is one of the major livestock production systems in the South Western part of Ethiopia [2].The breeds make immense contributions which are both tangible and intangible in nature. Some of the tangible benefits of sheep are immediate cash income, meat, milk, skin, and manure while the intangible benefit includes social prestige among the community members. Moreover, sheep play great role in the economy of the country by being exportable items and thus, are sources of much needed foreign currency [3].

Bonga sheep, one of the well-known and largest breed of Ethiopia, is characterized by long and wide fat tail with tapering and twisted end, both male and female are polled, short and smooth hair, mainly convex facial profile of male and predominantly light red coat color. The breed is known by its docile temperament, good fattening potential, fast growth and prolificacy [4].

To improve productivity of local breeds, crossbreeding based on imported sires has been followed for many years though with little success [5]. Therefore, improving locally adapted and diverse breeds through selective breeding has been considered as an option in developing countries to satisfy the growing demand for animal products [6]. Consequently, community-based breeding programs (CBBPs) were initiated in Ethiopia in 2009 by the International Centre for Agricultural Research in Dry Areas (ICARDA), the International Livestock Research Institute (ILRI) and the Austrian University of Natural Resources and Life Sciences (BOKU) in collaboration with the National and Regional Agricultural Research Systems in Ethiopia involving four breeds of sheep (Bonga, Afar, Horro, and Menz) [6]. The breeding programs for all breeds except Afar are still being implemented with active participation of the communities keeping the animals. For Afar; flock mobility, very high temperature, frequent droughts and poor infrastructure in the pastoral system; so far limits designing and implementation of community based breeding programs in pastoral areas [7]. Bonga CBBP being the most successful program in Ethiopia has been upscaled and additional CBBPs have been established [6].

Reproductive performances of sheep together with, survival and growth traits are important determinants of productivity [8]. The breeding objective traits identified by the community in Bonga CBBPs were growth rate, tail type, presence or absence of horn, twining rate, mothering ability and coat color [9]. For accurate genetic evaluation and selection, estimates of genetic parameters for traits of importance should be known [10]. In order to achieve the largest possible gains, a thorough evaluation of the program is needed [11]. Besides, it is a relevant tool to show that the promised benefits for farmers can be achieved and that livestock breeding is a sustainable intervention strategy under CBBP.

As has been indicated above, Bonga CBBP started in 2009 and through the upscale program an additional 14 CBBP were established since 2012. Frequent updating the genetic parameter estimate through evaluation is important and an integral component of breeding program. Therefore, the objectives of this study were to evaluate the genetic trend for reproductive traits and to estimate genetic parameters which help for optimization of the programs.

## MATERIALS AND METHODS

### Description of study area

The study was conducted in four districts, namely Adiyo, Gesha, Shisho-Ende, and Tello, of Kaffa zone of Southern Nation Nationalities People Region, Ethiopia. The area is characterized by mixed crop-livestock production system. It has one major rainy season that extends from May to October and a dry season that lasts from October to April [9]. The altitude range of study area is 1,600 to 3,348 meters above sea level and the minimum and maximum temperature was 14°C and 32°C with an average of 24°C. Similarly, the recorded minimum and maximum rain fall within the period of data 2012 to 2018 collections was 1,079 and 2,032 mm per year with an average of 1,617 mm/yr.

### Breeding program and animal management

Among the 14 CBBPs, five (Abeta, Buta, Dacha, Dirbedo, and Shosha) were established in 2012 while the remaining were during 2014. Selection of male lambs is being carried at two stages: screening of heavy weaners at weaning (3-month) followed by selection at six months of age (post weaning) by using their estimated breeding values (EBV). When the Bonga CBBP started, selection was carried out at six months. However, because of fast growth potential of the breed, it was noted that many lambs are sold before they reach the selection age. Therefore, the two-stage selection was implemented to keep the best ram lambs within the flock. Candidate lambs which had horn, short tail or black coat color were culled regardless of their EBVs.

Flocks being indoors at night in pens made up of bamboo walls and by any locally available corrugated roofing materials whereas, some farmers kept their flock around homestead at night. Flocks were tethered especially adult male and females during crop cultivation period. Therefore, main feed resource was pasture but additionally crop residue and kitchen leftovers were used. Feed availability and abundance vary with rainfall patterns. Comparatively huge amount of feed was available in the rain season whereas feed was less in both quality and quantity during the dry season.

### Data collection

Performance and pedigree data for this study were collected from the breeding database of out-scaled Bonga sheep CBBPs. The reproductive performance data used for this study included age at first lambing (AFL), lambing interval (LI), annual reproductive rate (ARR), and litter size (LS). Annual reproductive rate refers the number of young produced per breeding ewe female per year and it was calculated as: ARR = (365×LS)/LI. The detailed data structure and number of records for studied traits was indicated in Table 1.

### Data analysis

First, the data were checked for pedigree structure using pedigree viewer then Proc Univariate in Statistical Analysis System (SAS) [12] was employed prior to any analysis to check for analysis of variances assumptions. The phenotypic evaluation was done using the general linear model procedures of the SAS fitting non-genetic factors like year of birth (7 levels: 2012 through 2018), type of birth (3 level: single, twin, triple, and above), season of birth (2 level: wet and dry), dam parity (7 levels; parity 1 to 6, and 7 and above) and CBBP cooperative (13 levels) as fixed effect for AFL. Whereas lambing year, lambing season, lambing type, CBBP cooperatives and lambing parity at the same level with AFL considered for LI, LS, and ARR. Significant least square means were separated using Adjusted Tukey-Kramer method in SAS. All significant fixed effects were included in the genetic analysis.

Co(variance) components, genetic parameters and breeding values (EBVs) were estimated by restricted maximum likelihood fitting an animal model using WOMBAT software [13]. The following six univariate for AFL and repeatability for LI, LS, and ARR animal models were tested for each trait. The statistical models used were:

Model 1: y = **X_b_**+**Z_1a_**+e
Model 2: y = **X_b_**+**Z_1a_**+**Z_3pe_**+e
Model 3: y = **X_b_**+**Z_1a_**+**Z_2m_**+e with Cov(a,m) = 0
Model 4: y = **X_b_**+**Z_1a_**+**Z_2m_**+e with Cov(a,m) = Aσ_am_
Model 5: y = **X_b_**+**Z_1a_**+**Z_2m_**+**Z_3pe_**+e with Cov(a,m) = 0
Model 6: y = **X_b_**+**Z_1a_**+**Z_2m_**+**Z_3pe_**+e with Cov(a,m) = Aσ_am_

Where, y is n×1 vector of observations for each trait, b is a vector of fixed effects (year, parity, season, sex, CBBP cooperative and birth type), a, m, pe, and e are vector of random effects for direct additive genetic effects, maternal additive genetic effects, animal permanent environmental effect and residual effects, respectively. **X**, **Z_1_**, **Z_2_**, and **Z_3_** are the incidence matrices of fixed effect, direct additive genetic effect, maternal genetic effect and animal permanent environmental effect for LI, LS, and ARR but permanent environmental effect of the dam for AFL. A is the numerator relationship matrix between animals, and σ_am_ covariance between direct and maternal genetic effects. According to El Fadili et al [14] the (co)variance structure of the random effects were:

$$\begin{array}{l} {\text{V}}_{\left(\text{a}\right)}=\text{A}{\sigma^{2}}_{\text{a}},\;{\text{V}}_{\left(\text{m}\right)}=\text{A}{\sigma^{2}}_{\text{m}},\;{\text{V}}_{\left(\text{pe}\right)}={\text{I}}_{\text{d}}{\sigma^{2}}_{\text{pe}},\;{\text{V}}_{\left(\text{c}\right)}={\text{I}}_{\text{d}}{\sigma^{2}}_{\text{c}},\\ {\text{V}}_{\left(\text{e}\right)}={\text{I}}_{\text{n}}{\sigma^{2}}_{\text{e}},\text{Cov}(\text{a},\text{m})=\text{A}\sigma_{\text{am}} \end{array}$$

Where, σ_a_^2^, σ_m_^2^, σ_pe_^2^, σ_c_^2^, σ_am_, and σ_e_^2^ are direct additive genetic variance, maternal additive genetic variance, animal permanent environmental variance, maternal permanent environmental variance, direct-maternal genetic covariance, and residual variance, respectively. I_d_ and I_n_ are identity matrices of an order equal to the number of dams and the number of lambs, respectively.

Estimates of additive direct (h^2^_a_) and additive maternal (h^2^_m_) heritability, ratio of animal permanent environmental variance with phenotypic variance (pe^2^) and ratio of maternal permanent environmental variance with phenotypic variance (c^2^) were calculated as ratios of estimates of additive direct (σ_a_^2^), additive maternal (σ_m_^2^), animal permanent environmental (σ_pe_^2^), and maternal permanent environmental (σ_c_^2^) variances to the phenotypic variance (σ_p_^2^), respectively. Total heritability was calculated according to the following equation [15]:

$${{\text{h}}^{2}}_{\text{t}}=({\sigma^{2}}_{\text{a}}+0.5{\sigma^{2}}_{\text{m}}+1.5\sigma_{\text{am}})/{\sigma^{2}}_{\text{p}}$$

The genetic correlation between direct and maternal genetic effects (r_am_) was estimated as the ratio of the estimates of the σ_am_ to the product of the square roots of the estimates of σ^2^_a_ and σ^2^_m_ [16].

The genetic correlation (r_g_) between traits were estimated as the ratio of the estimates of the genetic covariance between the traits 1 and 2 to the product of the square roots of the estimates of genetic variance for trait 1 and genetic variance for trait 2.

Genetic trends of the traits were estimated by regression of predicted breeding values on the birth year [17]. Genetic gain was calculated as the difference between the EBVs of last and first year of the program [18].

Repeatability (r) was estimated according to Mokhtari et al [19]:

$$\text{r}=({\sigma^{2}}_{\text{a}}+{\sigma^{2}}_{\text{pe}})/{\sigma^{2}}_{\text{p}}$$

Where, σ^2^_a_ = additive genetic variance; σ^2^_pe_ = animal permanent environmental variance σ^2^_p_ = phenotypic variance.

To determine the most appropriate model likelihood ratio tests (LRT) was used. The significance of model comparison was done from univariate analysis of animal models with and without including the effects as a random effect and compared the final log-likelihoods (Maximum log L) by chi-square distribution for α = 0.05 with one degree of freedom [20]. An effect was considered to have a significant influence when its inclusion caused a significant increase in log likelihood, compared with the model in which it was ignored.

$${\chi^{2}}_{1\text{df}}=2[\text{L}{\left(x\right)}_{\text{f}}-\text{L}{\left(x\right)}_{\text{r}}]$$

The LRT was distributed as a χ^2^ statistic with degrees of freedom equal to (p_f_–p_r_). Where LRT = Log likelihood ratio test, L(**x**)_f_ = maximum likelihood for full model, L(**x**)_r_ = maximum likelihood for reduced model, P_f_ = number of parameter for full model, and P_r_ = number of parameter for reduced model. If the chi-square distribution value is significance at (p<0.05) the full model is best fit the data [20].

## RESULTS AND DISCUSSION

### Fixed effects

#### Age at first lambing

The overall least squares mean±standard error (SE) and coefficient of variation of AFL for Bonga ewes were 375±12.5 days and 19.8%, respectively (Table 2). Age at first lambing was significantly affected (p<0.05) by birth year but not by CBBP cooperatives, dam parity, birth season and birth type (Table 2). In the previous study by Edea et al [21] AFL of Bonga was 447±93 days but in the current study it was shorter. Age at first lambing was decreasing across years from 423±24.4 to 361±14.4 days. This indicates that the breeding program had shortened AFL due to selection of fast-growing rams and using them for breeding. According to Ayele and Urge [22] year of birth of lamb influenced AFL through its effect on feed supply and quality.

#### Lambing interval

The overall least squares mean±SE of LI of Bonga sheep was 284±9.9 days. It was influenced by lambing year, CBBP cooperative, dam parity and season of lambing (p<0.05), however, the influence of lambing type was non-significant (Table 2). The LI decreased from 302±11.6 days in 2012 to 272±5.5 days in 2017 indicating that selective breeding under CBBP yielded positive results. Kicho cooperative had a shorter LI than the others, which was 272±10.9 days and the longest was recorded from Guta cooperative, which was 295±11.4 days. The difference of LI across cooperative was mainly due to variation in the management activities like a quicker follow up of heat signs and feeding management. The LI was shorter in wet season (278±10.0 days) compared to dry season (289±10.0 days). The LI was longer in first parity ewes (295±10.1 days) whereas it was shorter in fifth parity ewes (273±11.3 days). The possible reason for longer LI in first parity dams may be ascribed to growing the dam resulted in poor development of reproductive system. The current result was comparable with 268±63.9 days reported by Edea et al [21] for the same breed (Bonga). Both AFL and LI are highly influenced by regular supervision for heat signs because the animals in the study area were mainly tethered on private land and mainly controlled breeding system was practiced.

#### Litter size

The overall least squares mean±SE of Bonga sheep was 1.45±0.010 and the coefficient of variation was 36.62%. The percentages of twins and above were 40.13%. Litter size was significantly influenced by CBBP cooperative and dam parity (p<0.001) but non-significant for lambing year and lambing season (Table 2). Perusal of LS in the cooperatives showed that Omashonga cooperative had highest LS (1.57±0.020) whereas the lowest (1.38±0.010) was observed in Dacha cooperative. The difference of LS across cooperatives was mainly due to variation in the management activities. In the study area multiple lambs were provided additional milk and milk products and suckling was controlled deliberately to avoid dominating either of the lambs. Ewes in the first parity had lowest (1.40±0.008) LS whereas ewes in ≥7 parity had highest (1.48±0.030) LS. The possible reason for lower LS in first parity dams may be their poorly developed reproductive systems due to their younger age.

#### Annual reproductive rate

The overall least squares mean± SE of ARR was 2.31±0.050 lambs/ewe/yr and the coefficient of variation was 38.84%. Annual reproductive rate was significantly affected by lambing year, CBBP cooperatives, dam parity and lambing season (p<0.01) (Table 2). The result of dam parity indicated that there was a gradual increase in ARR from 1.56±0.050 lambs/ewe/yr (First parity) to 2.67±0.080 lambs/ewe/yr (≥7 parity). This result was corresponding to the gradual increase in the LS in succeeding parities (Table 2).

### Model comparison

Importance of including both or one of each of additive maternal genetic or animal permanent environment effect on direct animal genetic effect was tested using LRT to determine the most appropriate model fitting the dataset (Table 3). Inclusion of both maternal additive genetic effect and animal permanent environmental effect (model 6) to direct animal genetic effect (model 1) significantly improved the log L for LS and ARR based on LRT distributed as chi-square test statistics (Table 3). However, for LI, inclusion of animal permanent environmental effect (model 2) significantly improved the log L but only additive genetic effect was the best fit for AFL (model 1) (Table 3).

### Genetic parameter estimation

#### (Co) variance components of reproductive traits

The estimates of (co)variance components and resulting genetic parameters for reproductive traits along with estimated maximum likelihood values for six models for each trait are presented in Table 3. Perusal of variance components of the best fitted model of each trait indicated that 389.22, 472.0, 0.04, and 0.13 of the total variations comprised of direct additive variance (σ^2^_a_) for AFL, LI, LS, and ARR, respectively. These values accounted for 6.57%, 6.38%, 16.66%, and 25% of the total variance indicating limited contribution of additive effect. The ratio of animal permanent environmental variance to phenotypic variance was higher for repeatable traits (LI, LS, and ARR) which were 0.51, 0.37, and 40, respectively. This indicated that improving the animals’ environment would result an improvement of these traits. This permanent effect is an environmental effect that contributes permanently to individual’s phenotype and is constant across repeated measures and which is not transmitted to across generation. For example intrauterine environment stimuli may impact foetal development and this permanently affects phenotype performance later in life [23].

#### Heritability estimation

Direct heritabilities from selected models for AFL, LI, LS, and ARR were 0.07±0.190, 0.06±0.120, 0.18±0.070, and 0.25±0.203, respectively. The heritability of LI and AFL (Table 3) was low indicating that these traits were influenced by environmental effects including ability of farmers to timely detect heat (Estrus), feeding system, and introduction of new ewes to the program and breeding sire using mechanism. Similarly, Maria et al [24] explained that low heritability estimates for these traits were expected. Direct genetic selection within the breed for AFL and LI may therefore not bring about much improvement. However, reproductive traits are aggregate traits and a small improvement in these traits would mean sizeable gain in terms of overall change in the other traits and is usually realized with simultaneous change in all components. Direct heritability of LS was higher than Ethiopian Horro sheep and other exotic sheep breeds [25,26]. Heritability estimate in the current study for AFL is lower than those reported for Brazilian Santa Ines sheep 0.13±0.10 [27] but comparable with 0.04± 0.017 reported for LI [27]. According to Maria et al [24] estimated 0.04 and 0.06 heritability for AFL and LI from multi-breed meat sheep population in Brazil. Average heritability for AFL and LI was low 0.07 and 0.02, respectively for Iranian Lori-Bakhtiari sheep [28].

#### Correlation estimate

Bivariate analysis revealed negative genetic correlation (−0.66±0.03) between AFL and LS but positive for phenotypic correlation (0.03±0.052). This indicated that a fast growing ewe lamb has the ability to achieve a higher prolificacy rate. The higher the ovulation rate, the more oocytes will be available for fertilization during the estrous and therefore increases the possibility of a larger litter [29]. Correlations between LI and LS were 0.844±0.850 (genetic) and 0.023±0.016 (phenotypic correlation). The high genetic correlation between LI and LS indicated that similar genetic factors influence these traits. This association showed that continuous reduction in LI result a reduction of LS or twining ability. Correlated response is expected between AFL and LS due to medium and negative correlation but not expected for LI and LS through improvement of either of the traits due their strong and positive correlation. Also, there was strong (0.959±0.116) genetic and 0.753±0.007 (phenotypic) correlation between LS and ARR (Table 4).

#### Repeatability (r) estimate

The estimate of repeatability of LI, LS, and ARR was 0.57, 0.54, and 0.65, respectively, for Bonga sheep. High repeatability indicates that culling of animals based on performance in single or only few initially available records could be done. The result was much higher than Horro sheep for LS 0.12 [25] and Pelibuey ewes of southeastern México 0.06±0.20 and 0.12±0.04 for LI and LS, respectively [30]. Higher repeatability than heritability estimates of a trait showed that the traits were influenced by non-additive genetic effects and permanent environmental effects, and to improve these traits one should improve environmental effects or management of flock in first step [31].

#### Genetic trends

**Figure 1:**
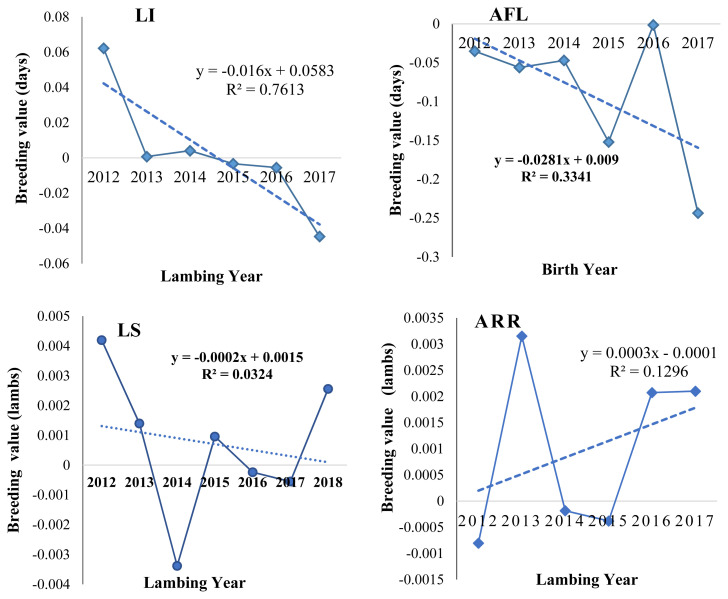
Means of predicted breeding value for different reproductive traits over years. Genetic improvement trend was estimated through regression of estimated breeding value across birth year for AFL and lambing year for LI, LS, and ARR. The graphs indicated that all evaluated reproductive traits were moving in the right direction except LS which was in a negative trend. LI, lambing interval; AFL, age at first lambing; LS, litter size; ARR, annual reproductive rate.

Genetic trends of different reproductive traits in Bonga sheep (Figure 1) were estimated using regression of the average predicted breeding values obtained from the best fitted model for each trait on year of birth for AFL and year of lambing for LI and LS traits. The annual genetic decreasing value for each reproductive trait were −0.0281 days, −0.016 days and −0.0002 lambs for AFL, LI, and LS, respectively whereas positive 0.0003 lambs/ewe/yr for ARR. Statistically all reproductive traits annual genetic trend was not significance. The breeder aims to reduce both AFL and LI and negatively directed EBV for these traits was in the right direction. However, for LS the breeder aims to increase twining rate but negative EBV caused decrease in LS. The possible reason for this was more emphasis for improving body weight due to selection of higher body weight sires born in single for breeding service, negatively correlated between body weights with LS and reduction in LI due to them being positively correlated. Therefore, there needs to be special attention for either on-farm or on-station conservation for prolific flocks. Positive and better annual genetic trend for LS was estimated from Horro sheep 0.0009±0.004 [32]. Related annual trend of AFL −0.012 days/yr and LS −0.0003 lambs/yr was recorded from Brazilian Santa Ines sheep [27].

## IMPLICATIONS

Difference in heritability estimates obtained from the different models suggests that model choice is an important aspect for obtaining reliable parameter estimates to be used in prediction of breeding values. Heritability estimates for age at first lambing and lambing interval were low indicating difficulty of improving these traits through direct selection because these traits are highly influenced by environment. Therefore, improvement of such traits could be made through manipulation of production management to reduce environmental influence. The possible reason for negative trend of litter size may be a greater emphasis for improving body weight and litter size being negatively correlated with body weight. Therefore, this trait needs special attention for either on-farm or on-station conservation for prolific flocks.

## Figures and Tables

**Table 1:** Pedigree structure of Bonga ewes included in the study

Item	N
No. of animals	22,214
No. of records	11,629
No. of sires	240
No. of dams	768
No. of dams with records and progeny in data	768
No. of animals with unknown sire	21,472
No. of animals with unknown dam	20,975
No. of animals with both parents’ unknown	20,972
No. of animals w/out offspring	21,206
No. of animals with offspring	1,008
No. of animals with known maternal grandsire	68
No. of animals with known maternal grand dam	122

**Table 2:** Least square means of reproductive traits (±standard error) influenced by different fixed effects

Fixed effect	N	AFL	Fixed effect	N	LI	N	LS	N	ARR
	
**Overall**	**412**	**375±12.5**	**Overall**	**3,841**	**283±9.9**	**11,629**	**1.45±0.010**	**3,841**	**2.31±0.050**
CV %		19.8	CV %		30.5		36.62		38.84
Range		255–540	Range		170 – 539		1–4		0.68 – 5.56
**Birth year**		^*^	**Lambing year**		^*^		NS		^***^
** ** **Coop**		NS	** ** **Coop**		^*^		^***^		^***^
Abeta	62	389±15.6	Abeta	486	275±10.5^ab^	1,292	1.42±0.010^bc^	486	2.21±0.060^ab^
A.geta	39	389±17.2	A.geta	270	287±11.2^ab^	1,046	1.48±0.01^b^	270	2.38±0.060^a^
A.kola	15	376±24.1	A.kola	95	273±13.4^ab^	451	1.46±0.020^bc^	95	2.48±0.080^a^
Dacha	45	380±17.1	Dacha	471	282±10.5^ab^	1,229	1.38±0.010^c^	471	2.14±0.060^b^
Didifa	17	370±23.1	Didifa	240	292±11.2^ab^	767	1.44±0.020^bc^	240	2.23±0.060^ab^
D.bedo	32	359±17.9	D.bedo	354	284±10.8^ab^	1,182	1.42±0.010^bc^	354	2.29±0.060^ab^
Guta	22	400±21.3	Guta	301	295±11.4^b^	844	1.39±0.010^c^	301	2.19±0.060^ab^
Kicho	41	345±17.5	Kicho	317	272±10.9^a^	761	1.45±0.020^bc^	317	2.43±0.060^a^
Meduta	47	372±16.2	Meduta	380	280±10.8^ab^	1,011	1.43±0.020^bc^	380	2.30±0.060^ab^
O.honga	14	364±24.1	O.honga	203	287±11.5^ab^	591	1.57±0.020^a^	203	2.42±0.060^a^
Shosha	27	376±18.5	Shosha	355	288±10.8^ab^	1,168	1.47±0.010^b^	355	2.31±0.060^ab^
W.lla	33	382±18.4	W.lla	172	294±11.9^ab^	551	1.48±0.020^bc^	172	2.25±0.060^ab^
Yama	18	375±22.1	Yama	197	277±11.6^ab^	736	1.43±0.020^bc^	197	2.34±0.060^ab^
**Birth season**		NS	**Lambing season**		^***^		NS		^**^
Dry	182	372±12.8	Dry	1,760	289±10.0	5,665	1.45±0.009	1,760	2.27±0.050
Wet	230	378±13.4	Wet	2,081	278±10.0	5,964	1.44±0.007	2,081	2.34±0.050
**Birth type**		NS	**Lambing type**		NS		NA		NA
Single	203	377±11.3	Single	2,355	278±9.4				
Twin	196	387±11.0	Twin	1,437	282±9.2				
≥Triple	13	362±24.3	≥Triple	49	291±15.3				
**Dam parity**		NS	**Dam parity**		^*^		^***^		^***^
1	110	370±14.1	1	1,411	295±10.1^b^	3,193	1.40±0.008^b^	1,411	1.56±0.050^d^
2	124	367±13.7	2	1,073	288±10.1^ab^	3,160	1.42±0.009^ab^	1,073	2.30±0.050^bc^
3	92	372±13.9	3	563	289±10.2^ab^	2,908	1.42±0.010^ab^	563	2.21±0.060^c^
4	35	380±17.3	4	381	288±10.7^ab^	956	1.48±0.010^a^	381	2.34±0.060^bc^
5	23	379±19.5	5	199	273±11.3^a^	583	1.46±0.010^a^	199	2.46±0.060^ab^
6	20	379±20.7	6	134	275±12.3^ab^	407	1.46±0.020^ab^	134	2.61±0.070^a^
≥ 7	8	381±30.4	≥7	80	277±13.7^ab^	422	1.48±0.030^a^	80	2.67±0.080^a^

AFL, age at first lambing; LI, lambing interval; ARR, annual reproductive rate; LS, litter size; Coop, CBBP cooperatives; B.type, birth type; A.geta, Alargeta; A.kola, Angiokola; D.bedo, Dirbedo; O.honga, Omashonga; W.lla, Wanabolla; NA, not applicable.

a–d Means with different letter in column within fixed effects are significantly different;

* p<0.05,

** p<0.01, and

*** p<0.001

NS, non-significance.

**Table 3:** Estimates of (co)variance components and genetic parameter for reproductive traits from univariate and repeatability animal model analyses

Trait	M1	M2	M3	M4	M5	M6
Age at first lambing						
σ^2^_a_	**389.2**	90.1	0.2	332.9	0.1	332.1
σ^2^_m_	-	-	1,277.9	2,354.9	1,148.7	2,357.7
σ^2^_e_	**5,538.4**	4,621.0	4,653.8	4,107.8	4,598.4	4,108.1
σ^2^_c_	-	1,222.3	-	-	185.5	0.1468
σ^2^_p_	**5,927.6**	5,933.4	5,931.8	5,914.7	5,932.7	5,914.5
σ_am_	-	-	-	−881.02	-	−883.6
h^2^_a_	**0.07±0.190**	0.02±0.200	0.00±0.200	0.06±0.300	0.00±0.200	0.06±0.300
h^2^_m_	-	-	0.22±0.2	0.4±0.3	0.19±0.400	0.40±0.900
c^2^	-	0.20±0.200	-	-	0.03±0.400	0.00±0.500
h^2^_t_	**0.07**	0.02	0.11	0.03	0.10	0.03
r_am_	-	-	-	−0.99	-	−0.99
log L	**−1,973.92**	−1,973.55	−1,973.33	−1,973.01	−1,973.33	−1,973.01
LRT	**0.390945**	-	0.422987	-	0.422266	0.390945
Lambing interval						
σ^2^_a_	467.8	**472.0**	471.6	948.3	462.8	891.7
σ^2^_m_	-	-	0.03	59.909	0.02	45.94
σ^2^_e_	6,927.7	**3,147.1**	6,924.0	6,412.6	6,302.5	3,236.8
σ^2^_pe_	-	**3,776.5**	-	-	630.25	3,236.8
σ^2^_p_	7395.6	**7,395.6**	7,395.6	7,182.4	7,395.5	7,208.8
σ_am_	-	-	-	−238.34	-	−202.40
h^2^_a_	0.06±0.100	**0.06±0.100**	0.06±0.100	0.13±0.200	0.06±0.100	0.12±0.200
h^2^_m_	-	-	0.00±0.100	0.01±0.100	0.00±0.1	0.01±0.100
pe^2^	-	**0.51±0.100**	-	-	0.09±0.002	0.45±0.200
h^2^_t_	0.06	**0.06**	0.06	0.09	0.06	0.08
r_am_	-	-	-	−1	-	−1
r	-	**0.57**	-	-	-	-
log L	−18,969.7	**−18,969.7**	−18,969.7	−18,969.6	−18,969.7	−18,969.6
LRT	1	-	0.67834	-	0.67834	1
Litter size						
σ^2^_a_	0.02	0.02	0.01	0.04	0.01	**0.04**
σ^2^_m_	-	-	0.03	0.03	0.03	**0.03**
σ^2^_e_	0.24	0.12	0.21	0.18	0.10	**0.09**
σ^2^_pe_	-	0.12	-	-	0.10	**0.09**
σ^2^_p_	0.26	0.26	0.26	0.24	0.26	**0.24**
σ_am_	-	-	-	−0.02	-	**−0.02**
h^2^_a_	0.09±0.040	0.09±0.040	0.06±0.050	0.18±0.070	0.06±0.050	**0.18±0.070**
h^2^_m_	-	-	0.12±0.030	0.15±0.030	0.12±0.030	**0.15±0.030**
pe^2^	-	0.45±0.040	-	-	0.24±0.003	**0.37±0.007**
h^2^_t_	0.09	0.09	0.09	0.10	0.09	**0.10**
r_am_	-	-	-	−0.53±0.080	-	**−0.53±0.080**
r	-	0.54	-	-	-	**0.54**
log L	1,768.0	1,768.0	1,771.4	1774.6	1,771.4	**1,774.6**
LRT	1	-	0.010488	-	0.010488	**1**
Annual reproductive rate						
σ^2^_a_	0.057	0.006	0.001	0.13	0.006	**0.13**
σ^2^_m_	-	-	0.001	0.05346	0.009	**0.05**
σ^2^_e_	0.57	0.30	0.63	0.41759	0.29	**0.21**
σ^2^_pe_	-	0.30	-	-	0.29	**0.21**
σ^2^_p_	0.63	0.61	0.63	0.52	0.60	**0.52**
σ_am_		-	-	−0.08	-	−**0.08**
h^2^_a_	0.09±0.126	0.01±0.114	0.002±0.13	0.26±0.203	0.009±0.13	**0.25±0.203**
h^2^_m_	-	-	0.002±0.09	0.10±0.099	0.02±0.095	**0.10±0.099**
pe^2^	-	0.49±0.115	-	-	0.49±0.011	**0.40±0.029**
h^2^_t_	0.09	0.01	0.002	0.07	0.02	**0.06**
r_am_	-	-	-	−0.99±0.354	-	**−0.991±0.35**
r	-					**0.65**
Log L	−987.9	−985.0	−987.5	−982.7	−984.79	**−982.7**
LRT	0.016545	-	0.001997	-	0.040663	**0.016545**

M1, M2, M3, M4, M5, and M6: model 1, 2, 3, 4, 5, and 6; σ^2^_a_, σ^2^_m_, σ^2^_e_, σ^2^_c_, σ^2^_pe_, σ^2^_p_: variance of direct, maternal, residual, maternal permanent environment, animal permanent environment and phenotypic, respectively; σ_am_, covariance between direct and maternal; h^2^_a_, h^2^_m_, h^2^_t_ heritability of direct, maternal and total, respectively; c^2^, ratio of maternal permanent environmental variance to phenotypic variance; pe^2^, ratio of animal permanent environment to phenotypic variance; r_am_, genetic correlation between direct and maternal; r, repeatability; log L, maximum log likelihood; LRT, likelihood ratio test *X*^2^ chi-square test value; hyphen (−) indicate for equal number of parameter models.

**Table 4:** Direct additive genetic below diagonal and phenotypic above diagonal correlation±standard error of reproductive traits

Traits	AFL	LI	LS	ARR
AFL	-	0.002±0.000	0.030±0.052	0.100±0.001
LI	0.002±0.012	-	0.023±0.016	0.036±0.001
LS	−0.660±0.03	0.844±0.850	-	0.753±0.007
ARR	0.100±1.000	0.036±0.009	0.959±0.116	-

AFL, age at first lambing; LI, lambing interval; LS, litter size; ARR, annual reproductive rate.
